# Gut Microbiota Dysbiosis–Immune Hyperresponse–Inflammation Triad in Coronavirus Disease 2019 (COVID-19): Impact of Pharmacological and Nutraceutical Approaches

**DOI:** 10.3390/microorganisms8101514

**Published:** 2020-10-01

**Authors:** Carolina Ferreira, Sofia D. Viana, Flávio Reis

**Affiliations:** 1 Institute of Pharmacology & Experimental Therapeutics, Coimbra Institute for Clinical and Biomedical Research (iCBR), Faculty of Medicine, University of Coimbra, 3000-548 Coimbra, Portugal; f202carolina@gmail.com; 2 Center for Innovative Biomedicine and Biotechnology (CIBB), University of Coimbra, 3004-504 Coimbra, Portugal; 3 Clinical Academic Center of Coimbra (CACC), 3000-075 Coimbra, Portugal; 4 Polytechnic Institute of Coimbra, ESTESC-Coimbra Health School, Pharmacy, 3046-854 Coimbra, Portugal

**Keywords:** COVID-19, SARS-CoV-2 infection, gut microbiota dysbiosis, immune hyperresponse, inflammation, pharmacological and nutraceutical approaches

## Abstract

Coronavirus Disease 2019 (COVID-19) is a pandemic infection caused by a novel coronavirus named severe acute respiratory syndrome coronavirus 2 (SARS-CoV-2). Patients present a complex clinical picture that, in severe cases, evolves to respiratory, hepatic, gastrointestinal, and neurological complications, and eventually death. The underlying pathophysiological mechanisms are complex and multifactorial and have been summarized as a hyperresponse of the immune system that originates an inflammatory/cytokine storm. In elderly patients, particularly in those with pre-existing cardiovascular, metabolic, renal, and pulmonary disorders, the disease is particularly severe, causing prolonged hospitalization at intensive care units (ICU) and an increased mortality rate. Curiously, the same populations have been described as more prone to a gut microbiota (GM) dysbiosis profile. Intestinal microflora plays a major role in many metabolic and immune functions of the host, including to educate and strengthen the immune system to fight infections, namely of viral origin. Notably, recent studies suggest the existence of GM dysbiosis in COVID-19 patients. This review article highlights the interplay between the triad GM dysbiosis–immune hyperresponse–inflammation in the individual resilience/fragility to SARS-CoV-2 infection and presents the putative impact of pharmacological and nutraceutical approaches on the triumvirate, with focus on GM.

## 1. Introduction

In December of 2019, a cluster of pneumonia cases was detected in Wuhan, China, and it was confirmed that these cases were caused by infections from a novel coronavirus (CoV) named severe acute respiratory syndrome coronavirus 2 (SARS-CoV-2) [1]. SARS-CoV-2 is an enveloped non-segmented positive-sense RNA β-coronavirus [2,3] that presents high homology with two other coronaviruses: SARS-CoV, which broke out in 2002 in China; and the Middle East respiratory syndrome coronavirus (MERS-CoV), which surged in Saudi Arabia in 2012, both causing severe respiratory tract infections [4]. SARS-CoV-2 infects human cells after linking to angiotensin converting enzyme 2 (ACE2), causing a new disease named by the World Health Organization (WHO) as Coronavirus Disease 2019 (abbreviated as COVID-19), which was declared a pandemic on 11 March 2020 [5]. In less than 6 months, the infection spread all over the world, affecting more than 27 million people from almost all countries and causing almost 1 million deaths, as of September 2020.

The clinical manifestations resulting from the SARS-CoV-2 infection include fever, cough, fatigue, sputum production, shortness of breath, sore throat, headache, and in some cases gastrointestinal symptoms such as diarrhea and vomiting [1]. In most severe forms, COVID-19 patients often display several complications, including respiratory, hepatic, renal, cardiac, gastrointestinal, and neurological complications, which culminate in hospitalization and eventually in death [6]. Poor outcomes, represented by prolonged hospitalization at intensive care units (ICU) and increased mortality rate, have been obtained in elderly patients, particularly in those with pre-existing cardiovascular, metabolic, and renal disorders [7,8,9,10,11,12,13,14,15,16]. It has been reported that the frequency of cardio-cerebrovascular diseases, hypertension, and diabetes in infected patients who received care in the ICU could be three-, two-, and twofold higher, respectively, than counterparts receiving non-ICU care [11]. In these patients, the disease has been described as an inflammatory/cytokine storm that an ill-suited immune system is unable to manage [4,17]. Coherently, previous studies on SARS-CoV and MERS-CoV infections have shown an identical profile with hyperactivation of the immune system and subsequent exacerbated systemic inflammatory response [18]. This condition is characterized by an uncontrolled production of pro-inflammatory cytokines and can lead to organ failure and acute respiratory distress syndrome (ARDS) [4], a major complication of COVID-19 as well.

Interestingly, the same type of populations/diseases has been associated with a condition known as gut microbiota (GM) dysbiosis, characterized by impaired diversity and/or function of intestinal microflora [19,20]. Apart from relevant metabolic functions for the host homeostasis, GM exerts protective actions against pathogenic colonization of bacteria and virus, which could be at least partially attributed to their role in educating and strengthening the immune system [21]. There is evidence that SARS-CoV-2-infected individuals present deregulated GM [22,23], which might contribute to the poor outcomes in COVID-19 patients with pre-existing diseases influenced by GM dysbiosis, namely, cardiovascular, metabolic, and renal disorders. In this sense, therapeutic and nutraceutical interventions able to promote microbiota symbiosis, reinforce the immune system, and exert anti-inflammatory actions could be potentially useful.

Despite the efforts and investment made in research and development in recent months, there is still no vaccine or an effective treatment for this disease. However, a variety of options have been tested. This article highlights the GM dysbiosis–immune hyperresponse–inflammation triad that takes place in COVID-19. In addition, it provides a comprehensive review about the complex interplay between drugs and/or nutraceutical interventions with GM composition, intestinal mucosa, and host immune functions in the frame of COVID-19 therapeutic approaches currently under evaluation.

## 2. Main Features of SARS-CoV-2 Infection

Coronaviruses belong to the subgenus Sarbecovirus, Betacoronavirus genus of the subfamily Orthocoronavirinae, in the family Coronaviridae of the order Nidovirales. Orthocoronavirinae subfamily is further divided into four genera: alpha-coronavirus (α-CoV), beta-coronavirus (β-CoV), gamma-coronavirus (γ-CoV), and delta-coronavirus (δ-CoV) [4]. SARS-CoV-2 is a β-coronavirus, like the aforementioned SARS-CoV and MERS [4].

SARS-CoV-2 is spherically shaped and has spike projections on its surface [24]. This morphology makes the virus look like a crown, hence the name coronavirus, which conjugates the words corona (Latin for crown) and virus. It is an enveloped non-segmented positive-sense RNA virus with near 30 kb [2]. SARS-CoV-2′s genome encodes four structural proteins essential to the virus assembly and infection: spike surface glycoprotein (S), envelope protein (E), membrane protein (M), and nucleocapsid protein (N) [2]. S-glycoprotein is paramount for the virus attachment to the host cells and is cleaved via acid-dependent proteolysis by host proteases (e.g., cathepsin, transmembrane serine protease 2 (TMPRRS2), or furin protease) into two subunits, N-terminal S1 and membrane-bound C-terminal S2 [2,3]. S protein cleavage occurs upon viral interaction with ACE2 receptors expressed on human cells’ surface. While S1 subunit determines the virus–host range as well as the cellular tropism due to its receptor binding domain (RBD), S2 subunit mediates the viral fusion to the cell membrane [2]. In humans, viral replication occurs in distinct cell types on the basis of the ubiquitous expression of ACE2 receptors (e.g., lung cells, esophageal cells, epithelial cells, and enterocytes). This observation contributes to explain why COVID-19 is a multi-tissue infection rather than being merely restricted to the lower respiratory tract [3].

This virus presents a mean incubation interval ranging from 2 to 14 days, which implies a long transmission period [4]. Human-to-human SARS-CoV-2 transmission occurs mainly between family members and friends with close contact with patients or carriers, which is in contrast with the nosocomial transmission characterizing SARS-CoV and MERS-CoV [1]. SARS-CoV-2 can be transmitted between humans through respiratory droplets [4], but the respiratory tract might not be the only transmission course. Close contact could also compose a transmission route, for example, through direct or indirect contact with mucous membranes in the nose, mouth, or eyes [1,4]. It is acknowledged that asymptomatic individuals can transmit the virus, a feature that differentiates SARS-CoV-2 from SARS-CoV, as the latter was unable to elicit secondary infection during the incubation period [4].

Ultimately, SARS-CoV-2 possesses intrinsic genetic variability and is characterized by a high mutation rate, analogous to other RNA viruses. It is thought that any genetic adaptation in the virus sequence that eases human-to-human transmission might increase its virulence [25]. In fact, recent research suggests that mutations occurring in SARS-CoV-2 NSP2 and NSP3 proteins influence its differentiation mechanisms and infectious ability [1]. Tang et al. detected two different types of SARS-CoV-2, S- and L-type, the latter more aggressive and contagious [26]. Yet, these mutations may culminate in a phenomenon known as Muller’s ratchet, a process in which the absence of genetic recombination, especially in asexual populations, results in the accumulation of deleterious mutations in an irreversible manner [25,27]. This event can significantly reduce viral fitness and SARS-CoV-2 may become less virulent [25], hopefully in the near future.

## 3. GM Dysbiosis–Immune Hyperresponse–Inflammation Triad in COVID-19

For the past decades, studies of human microbial environments have been particularly focused on the human gut, mainly owing to important interplays between intestinal microbes and the host in distinct phenotypes of clinical relevance [28]. Humans (and other mammals) are inhabited by a complex gastrointestinal microbiota that includes distinct domains of life (e.g., bacteria and eukaryota). The host animal should then be envisaged as an extraordinary multispecies hybrid structure comprising a symbiotic ecosystem of eukaryotic and microbial cells with a mutually beneficial relationship [29].

The composition of intestinal flora is highly dynamic from birth onwards and is shaped by distinct environmental factors (e.g., diet, pre-/probiotics, drugs), resulting in a variety of microbial-host functions [30,31]. Human gastrointestinal tract (GIT) contains up to 2000 bacterial species, classified in 12 different phyla, where the most prevalent (>90%) are the *Proteobacteria* (Gram-negative), *Firmicutes* (Gram-positive), *Actinobacteria* (Gram-positive), and *Bacteroidetes* (Gram-negative) phyla [32,33]. Yet, increasing efforts to develop a deep understanding of gut microbiological environment diversity led to new emerging concepts, namely the existence of a “gut virome” as a key component of the healthy human microbiota [34]. The lack of universal phylogenetic markers in viruses (e.g., 16S rRNA in bacteria) makes virome studies challenging and cutting-edges approaches, such as large-scale metagenomic sequencing (MGS) analysis, are often mandatory. Human gut virome, composed by both prokaryotic (mostly bacterial) and eukaryotic (mostly human) viruses, is believed to share important information with all microbial components and may influence overall human health by molding gut community structure and function [35,36]. For instance, cooperative transmission of genes between bacteriophages (accounting for about 90% of the gut virome) and the infected bacteria may help the host to endure oxidative stress and antibiotic use [34,37]. It is now recognized that the gut virome composition is also extremely dynamic after birth. When compared with intestinal prokaryotic viruses, gut eukaryotic viruses are at very low abundance, but their almost ubiquitous presence hints for important functional significances in the host [38]. Interestingly, gut benign eukaryotic viruses acquired in early childhood have also been suggested to play a role in host immune maturation and several metabolic developments [39]. Yet, the presence of gut eukaryotic viruses is mainly documented during viral gastrointestinal infections such as Norwalk, Rotaviruses, Enteroviruses (the well-known agents of gastroenteritis in man), and more recently SARS-CoV-2 enteric infection [34].

Contemporary data highlight the ability of SARS-CoV-2 to generate newly produced virus progeny in infected human intestinal epithelial cells [40]. Accordingly, viral RNA was detected in feces from COVID-19 patients, similar to what has been reported in previous CoV outbreaks [41,42]. Even though SARS-CoV-2 fecal-oral transmission is still an open debate, accumulating evidence points to GIT as a battleground for continual viral replication as ACE-2 receptors are highly expressed in the intestinal epithelium [43,44]. Accordingly, high-throughput 16S rRNA gene and shotgun MGS analyses have shown significant alterations in the gut bacteriome of hospitalized COVID-19 patients, characterized by a significant decrease in GM diversity, depletion of beneficial bacterial symbionts, and enrichment of opportunistic pathogens (e.g., Streptococcus, Rothia, Actinomyces), which may correlate with the gastrointestinal complaints (e.g., abdominal pain, nausea, vomiting, diarrhoea) of the acute phase of the infection [22,23]. Interestingly, these symptoms often occur in the onset of the infection and may precede respiratory complaints [45,46,47]. Taking into account that ACE2 is able to regulate nutrient absorption and intestinal inflammation, it is currently postulated that enteric SARS-COV-2 infection and ACE2 imbalance may cause the gastroenteritis-like symptoms, intestinal homeostasis disruption, and GM dysbiosis observed in COVID-19 patients [22,47,48]. Likewise, several clinical trials are ongoing aiming to deeply characterize GM composition in COVID-19 patients and evaluate the putative crosstalk between GM and the disease outcome, as summarized in Table 1. Yet, the interaction between COVID-19 and intestinal microorganisms is not fully understood and similar studies are still warranted to assess the likelihood imbalance of gut virome upon enteric SARS-CoV-2 infection as well.

Noteworthy, the aforesaid shift in fecal microbiota composition was associated with COVID-19 severity and persisted even after SARS-CoV-2 clearance and respiratory symptoms resolution, hinting at long-term functional consequences of enteric SARS-CoV-2 infection to the host [23]. Conversely, a higher risk of infection and a faster and severe disease progression of COVID-19 cases is linked with pre-existing age-related pathologies (e.g., cardiovascular, metabolic, renal diseases) that display GM dysbiosis as a common denominator [7,8,9,10,11,12,13,14,15,16]. In fact, COVID-19 susceptibility and mortality rate is higher in elderly patients that tend to display a less-diverse GM profile, gut leakiness, and chronic low-grade inflammation [48,49,50]. Hence, a bidirectional relationship between SARS-CoV-2 infection and GM dysbiosis seems to take place, not only as a consequence of SARS-CoV-2 infection, but also as a putative cause/risk factor of COVID-19 poor outcomes. Accordingly, the concept of intestinal microbiota and SARS-CoV-2 interactome has been acknowledged as an important component to the susceptibility and severity of the infection [51].

One of the striking features of COVID-19 is the huge differences between the clinical outcomes of SARS-CoV-2 infection; while some patients are asymptomatic, others get seriously ill and, ultimately, die. Besides pre-existing pathologic conditions, factors like genetics, lifestyle, and environment may contribute to disease severity. Interestingly, these same factors are also key in GM shaping. Thus, SARS-CoV-2–gut interactions can trigger singular protective or deleterious microbiome pathways [51], adding an extra level of complexity to the individual suppression or promotion of SARS-CoV-2 viral infection. Among several mechanisms through which GM is able to impact COVID-19 outcomes, its intricate influence on host immunity deserves particular attention. In fact, it is generally established that commensal microbiota is paramount in assembling host immune effectors for an optimal antiviral immune response [52]. Both pathogenic and protective effects are elicited by intestinal microbes, depending on specific effectors (collectively termed microbe-associated molecular patterns, MAMPs) that engage with pattern recognition receptors (PRRs) towards a balanced tuning of innate and adaptive host immune responses and subsequent pro- and/or anti-inflammatory profile [53]. For instance, antigens derived from commensal organisms promote Treg cells’ differentiation following their presentation to naïve T cells by dendritic cells, prompting the secretion of anti-inflammatory cytokines (e.g., IL-10) and propagating local and systemic tolerance [29,54]. In addition, commensal bacteria are also able to stimulate goblet cell differentiation and the synthesis of the protective mucosal mucus layer [29,55]. Moreover, gut-associated lymphoid tissue (GALT, the bulk of immune surveillance and defense) development is strictly dependent on specific commensal microbial signals [56]. Conversely, pro-inflammatory immune responses may arise from pathogenic bacteria due to naïve T cells differentiation into Th1 and Th17 cells. Under such circumstances, an increased translocation of bacterial components across the gut epithelium may occur, leading to gut epithelium damage and, ultimately, gut–blood barrier disruption and systemic endotoxemia. These events are very likely to further compromise the host’s immune responses towards a long-lasting systemic pro-inflammatory setting [29,31]. Altogether, a strong interconnection between gut dysbiosis, immune system imbalance, and mucosal and systemic inflammation is clearly outlined. Noticeably, this triad is in motion in COVID-19 patients.

As a matter of fact, accumulating evidence flaunts an atypical immune over reactivity and a strong inflammatory scenario tagged as a “cytokine storm” or hypercytokinemia in COVID-19 victims. Most patients present lymphocytopenia, decreased leucocyte, and total T cells counts [1]. Furthermore, surviving T cells are functionally exhausted [1] and an increase in numerous inflammatory factors’ levels, including several interleukins (IL-2, IL-6, IL-7, and IL-10), tumor necrosis factor-α (TNF-α), granulocyte colony-stimulating factor (GCSF), macrophage inflammatory protein 1-α (MIP-1α), monocyte chemoattractant protein-1 (MCP-1), and 10 kD interferon gamma-induced protein (IP-10), is observed in patients with severe infection. On the basis of host enterocytes continuous SARS-CoV-2 replication, GM dysbiosis, and hyperresponsive immune system towards systemic inflammation, future studies are definitively required to disclose the plausible role of intestinal microflora in dictating the individual resilience or fragility to SARS-CoV-2 infection.

## 4. Therapeutic Opportunities for COVID-19 with Impact on the Triad and a Focus on GM

Unique patient characteristics (like a fingerprint) such as individual microbiome can be key to determine not only the disease manifestation, but also the response to therapy. Interestingly, a bidirectional relationship between GM and drugs is frequently outlined; while several drugs affect microflora, intestinal mucosa, and its barrier function, gut bacteriome can also modify the drug bioavailability to the host [57,58,59]. In addition, food is probably the most important single modulator of the intestinal microbiota throughout our entire lives, and there is also a strong influence of the intestinal flora on the digestion we make of the food, so that some components of our diet can only be processed by certain bacterial communities. In the following section, a comprehensive review is provided on the available information on the complex interplay between drugs and nutraceutical interventions with GM composition, intestinal mucosa, and host immune functions in the frame of COVID-19 therapeutic opportunities. It is clear that the list of therapeutic options that have been tested for COVID-19 treatment is much wider than the one discussed here. However, we chose to highlight interventions that have an unequivocal potential to interfere with the aforesaid triad, with a focus on GM.

### 4.1. Immunomodulatory and Anti-Inflammatory Drugs

#### 4.1.1. Chloroquine and Hydroxychloroquine

Antimalarial drugs have been identified as potential therapies for the treatment of COVID-19, in particular chloroquine (CQ) and hydroxychloroquine (HCQ), which are inexpensive drugs that have been used for a long time in the prevention and treatment of malaria and some autoimmune diseases (e.g., rheumatoid arthritis and lupus erythematosus) and have been tested in several trials for the treatment of COVID-19 patients [60]. CQ exhibits anti-inflammatory and immunosuppressive properties that could be useful to viral infections, thus explaining its experimental use to treat SARS and Zika, among others [61]. In fact, preclinical evidence showed that CQ was effective in inhibiting SARS-CoV infection by interfering with ACE2 [62]. There is also preclinical evidence that CQ, as well as HCQ, might inhibit SARS-CoV-2 infection by binding with high affinity to some cellular links (namely sialic acids and gangliosides), by inhibition of glycosylation of host receptors, proteolytic processing, and endosomal acidification, thus blocking virus entry [63]. It can also have immunomodulatory effects through inhibition of cytokine production, autophagy, and lysosomal activity in host cells [64,65]. Regarding clinical evidence, a systematic review on the efficacy and safety profile concluded that there is rationale, that is, evidence of effectiveness and of safety from long-term clinical use, for other indications that justify clinical research in COVID-19 patients. Nevertheless, the authors reinforce that better evidence of safety data and results from high-quality randomized clinical trials (RCTs) are urgently needed, recommending that the clinical use of CQ should either adhere to the monitored emergency use of unregistered interventions (MEURI) framework or be ethically approved as a trial according to WHO [66]. Although some clinical studies reported beneficial effects, namely, reduction of fever, improvement of lung imaging findings, and delaying of disease progression [67], there are no consistent data to conclude about a reduction of mortality by using CQ in COVID-19 patients. In addition, the potential side-effects, namely cardiac toxicity, and the possibility of drug–drug interaction, are major concerns [60]. With a structure similar to that of CQ, the use of HCQ (a 4-aminoquinoline) has been also tested in COVID-19 patients in several countries, with identical results and concerns [68]. Overall, and considering the better evidence up-to-date, owing to the risk of severe side-effects, as well as the inefficiency demonstrated by both CQ and HCQ in recent clinical trials with COVID-19 patients, health authorities, including the Federal Drug Administration (FDA) and WHO, did not recommend the use of these drugs.

The studies regarding the impact of CQ and HCQ on GM are scarce and the available preclinical and clinical data are dichotomous. In fact, in vitro HCQ increased the killing of *Escherichia coli* by Crohn’s disease monocyte-derived macrophages [69]. In vivo, HCQ treatment was able to adjust the impairment of GM caused by or related to rheumatoid arthritis-associated atherosclerosis in a high fat diet (HFD)-induced mice model [70]. Finally, patients diagnosed with Q fever endocarditis and treated with doxycycline and HCQ presented a reduced abundance of *Bacteroidetes*, *Firmicutes*, and *Lactobacillus*, which was associated with the treatment duration and eventually linked with the weight gain side effect found in those subjects [71]. However, the impact of both drugs on GM of COVID-19 patients, as well as on previous coronavirus infections, is lacking, thus limiting a better evaluation and recommending further research.

#### 4.1.2. Interferons

Interferons (IFNs) are a family of proteins secreted by cells of the immune system and can be divided in type 1, type 2, and type 3 IFNs [72]. All three types are cytokines produced in response to viral infections and are essential for regulating the immune response [73]. Type 1 IFNs are known for their capacity to directly induce an antiviral response in infected cells, as well as in those surrounding them, through the upregulation of molecules that antagonize the virus’ replication [73]. Moreover, type 1 IFNs are also essential for the activation of the innate immune response, namely natural killer (NK) cells [73]. These IFNs include the α, β, ε, ω, and κ subtypes [74]. On the other hand, type 2 IFNs, which include IFN-γ, are also a part of the innate immune response and are predominantly produced by NK cells during an infection [73].

Because of their immunomodulatory properties, type 1 IFNs are used in the treatment of several diseases and their administration in SARS-CoV and MERS-CoV cases has been extensively studied [74]. Chan et al. in vitro findings suggest that IFN-α and IFN-β present anti-MERS-CoV properties [75]. Falzanaro et al. demonstrated that the combination of IFN-α2b and ribavirin is capable of decreasing in vitro MERS-CoV replication [76]. Furthermore, Hart et al. tested several IFN subtypes, and demonstrated that, of all IFNs tested, IFN-β had the strongest effect in inhibiting the replication of the MERS-CoV virus [77]. Several studies suggest that treatment with distinct IFNs is effective in SARS-CoV infections. In fact, Hensley et al. reported that IFN-β1α might be efficient in the treatment of COVID-19 as it exhibits antiviral properties [78]. Cinatl et al. showed that IFNs inhibit SARS-CoV replication in vitro and IFN-β was the most potent one [79]. Furthermore, Morgenstern et al. reported that the combination of ribavirin and IFN-β inhibits SARS-CoV replication and might enable the reach of enough therapeutic plasma levels to suppress viral replication during the disease’s initial phase, reducing the risk of transmission [80]. IFN-γ does not show antiviral effects against SARS-CoV [81]. Regarding SARS-Cov-2, Lokugamage et al. demonstrated high susceptibility to IFN-α in vitro, which might be due to the virus’ Orf3b proteins losing their anti-interferon functions because they are truncated [82]. Moreover, Shen et al. showed that IFN-α2β sprays can reduce the rate of infection by SARS-CoV-2 [83]. A clinical trial demonstrated that recombinant human interferon alpha (rhIFNα) nasal drops can protect susceptible healthy people from infection, which indicates that IFNs might be a potential prevention option [84]. Furthermore, a clinical trial is currently taking place in order to access the efficacy and safety of IFN-α2β in the treatment of COVID-19 patients (NCT04293887).

There are several pieces of evidence suggesting that IFN signaling influences the crosstalk between the host and the microflora [72]. In mice, interferon tau supplementation caused marked changes on GM and expression of IL-17, including a decreased percentage of *Firmicutes* and increased of *Bacteroidetes* in the jejunum and ileum, in opposition to the changes on the colon and feces [85]. The authors concluded that interferon tau supplementation may affect metabolism through effects on GM. In mice, but with selective ablation of type 1 IFN signaling in the intestinal epithelium, major changes of microbiota composition were also obtained [86]. Another study showed that systemic influenza-induced type I IFN synthesis produces significant alterations in the GM profile [87]. In patients with multiple sclerosis receiving the disease-modifying drug IFN β-1b, microbiota composition is significantly altered towards the profile found in control subjects [88]. Unfortunately, data concerning the impact of IFNs in GM of COVID-19 patients are still lacking, but the rationale for this association exists, thus recommending improved research.

#### 4.1.3. Corticosteroids

Corticosteroids are steroid hormones with strong immunomodulatory and anti-inflammatory properties, which explain their use in the treatment of innumerous clinical pathologies, namely autoimmune and inflammatory diseases [89,90]. In addition, corticosteroids therapy is also used in the context of viral infections because of the above-mentioned effects [91,92], which are achieved by distinct mechanisms, including by their influence on the NF-kB and AP-1 signaling pathways, reducing the expression of pro-inflammatory genes and activating the transcription of anti-inflammatory ones [89,91].

Corticosteroid-based therapy was used in the treatment of the SARS outbreak in 2003 and has also been tested in COVID-19 patients [93,94,95]. However, the results have generated controversy and contradiction, with reports recommending both the use of corticosteroids-based treatments in COVID-19 patients and the opposite [96,97]. Zhou et al. reported that corticosteroids’ administration in short-term and moderate doses together with immunoglobulin significantly reduced lung damage and normalized body temperature, C-reactive protein levels, and the oxygenation index in COVID-19 patients [98]. Wu et al. found that administration of the synthetic glucocorticoid methylprednisolone in patients with COVID-19-caused ARDS led to a decrease in the risk of death by 62% and reduced the need for mechanical ventilation [99]. However, Liu et al. did not report any benefit from methylprednisolone administration in the short-term disease progression of the study’s participants [100]. Moreover, other studies have failed to provide conclusive evidence on the efficacy of corticosteroids in decreasing the mortality of ARDS. Furthermore, there are several studies demonstrating that corticosteroids have adverse effects in SARS-CoV-2 infected individuals. Zha et al. described a study where 11 out of 31 COVID-19 patients treated with corticosteroids showed more clinical symptoms, higher inflammation index, and chest CT abnormalities [92]. Overall, the results raised by the initial trials with corticosteroids were unable to fully encourage their use in COVID-19. In fact, because of the lack of efficacy of corticosteroids therapy, WHO does not recommend the use of these compounds unless there are alternative indications [101].

Remarkably, a recent study performed at Oxford University named RECOVERY trial (Randomised Evaluation of COVid-19 thERapY) brings new hope when it comes to the use of corticosteroids in the treatment of COVID-19 cases. The study’s results demonstrate that dexamethasone (DEX) might be a major breakthrough in the fight against SARS-CoV-2. DEX is a 9-fluoro-glucocorticoid with potent anti-inflammatory and immunosuppressive properties, being widely used to treat asthma, allergies, and autoimmune conditions [102]. By reducing cytokines synthesis, DEX is able to prevent the pulmonary and cardiac inflammation responsible for the serious respiratory problems observed in severe COVID-19 patients. The results of the Oxford study demonstrate that DEX decreases the risk of death in patients needing ventilation from 40% to 28%, and from 25% to 20% in patients needing oxygen. However, it does not seem to exert any effect in patients with mild symptoms and without respiratory issues. DEX is the first drug to reduce mortality from COVID-19 and presents the advantage of being instantly available and affordable worldwide, allowing populations from countries without universal health care to access treatment readily, which does not happen for newly developed drugs, which are often very expensive and have limited supply.

Besides their immunosuppressive properties, glucocorticoids also act as stress responses mediators [103,104]. The GIT and the immune system are particularly responsive to different stressors, having several cellular targets for stress mediators [104]. In fact, stress is associated with several gastrointestinal diseases and glucocorticoids therapy is used in the treatment of most of them [104]. Glucocorticoids influence important gut physiological functions, including mucosal integrity, intestinal permeability, and microbiota, as well as its interactions with the host [103,104]. It is now recognized that glucocorticoids might significantly affect the expression and function of mucin, the main constituent of the mucosal layer that conceals the intestinal epithelium [103]. Huang et al. showed that the GM of mice exposed to DEX suffered significant changes, in both acute and chronic treatment models [103]. In this study, DEX-treated mice presented higher levels of *Bifidobacterium* and *Lactobacillus*, bacteria associated with anti-inflammatory effects. On the other hand, genus *Mucispirillum*, which is known for its function as a mucin degrader, was absent in mice treated with DEX [103]. In a study performed by Wu et al., treatment with DEX resulted in a significant decrease of colonic microbiota richness and diversity, possibly through circadian rhythm disturbances and glucocorticoids receptors’ dysregulation, declining the relative abundances of *Firmicutes, Bacteroidetes, α-proteobacteria, γ-proteobacteria,* and *Actinobacteria* [105]. DEX-treated mice also showed decreased mucin secretion. On the other hand, treatment with DEX increased the levels of *Proteobacteria*, which is associated with a pro-inflammatory state. The same is seen for *Lactobacillus* and *Clostridiales*, which play important roles in the inflammation process. These results suggest that the enhanced inflammatory response in the colon may be due to DEX-induced changes in the abundance of these bacteria. Similar results were reported by Zhao and colleagues (2020), who observed that DEX administration elicited a decreased microbial richness and diversity [106]. Mice treated with DEX presented increased levels of *Butyriciococcus* and *Ruminoococcus*, which are butyrate-producing bacteria well-recognized for their protective role against certain diseases. Therefore, increased levels of these bacteria in the DEX-treated group might be associated, at least in part, with its therapeutic effects. On the other hand, *Lactobacillus*, which is associated with obesity and lower intestinal permeability, decreased in mice treated with DEX. Finally, not all studies available show an effect of DEX in the GM. In a study performed with goats [107], chronic exposure to DEX did not change intestinal microbiota composition, suggesting that the GM was highly resistant to treatment with DEX.

Although there are several lines of evidence on the influence of DEX in the GM composition, there is contradictory information on whether this influence has positive or negative consequences to the host. Therefore, further studies are necessary to determine the nature of the relationship between glucocorticoids in general, and DEX in particular, and GM.

### 4.2. Nutraceutical Approaches

#### 4.2.1. Prebiotics, Probiotics, and Synbiotics

According to the WHO, the International Scientific Association for Probiotics and Prebiotics (ISAPP), and the Food Agriculture Organization (FAO), probiotics are “live microorganisms that, when administered in adequate amounts, confer a health benefit on the host” [108,109], the most commonly used being *Lactobacillus* spp., *Bifidobacteria*, and *Saccharomyces* [108,109]. On the other hand, prebiotics are defined by WHO and FAO as “non-viable food components that confer health benefit(s) on the host associated with modulation of the microbiota” and may be used as an alternative to probiotics or as an additional support for them [110]. Prebiotics are mostly carbohydrate-based, non-digestible food ingredients that improve the host’s health by stimulating the growth and/or activity of specific colonic microorganisms [108,109,110]. Finally, synbiotics are a combination of probiotics and prebiotics administered together [111], improving the survival and implantation of live microbial dietary supplements in the GIT by stimulating the growth and/or activating the metabolism of health promoting bacteria [108].

The key function of these supplements is to restore the composition of the gut microbiome, ameliorating or preventing gut inflammation and other intestinal or systemic diseases [112]. They may also increase immune system efficacy and modulate pathogen-induced inflammation via toll-like receptor-regulated signaling pathways [110,113,114]. Furthermore, they can enhance vitamin absorption [110] and have also been shown to reduce the severity of certain allergic conditions [109].

Clinical evidence demonstrates that certain probiotic strains support the prevention of viral infections of the respiratory tract [109,113,115,116], as is the case of COVID-19. Particularly, studies show that patients on mechanical ventilation who were administered probiotics had significantly less ventilator-associated pneumonia [117,118]. Additionally, probiotic strains have been shown to modify the balance between proinflammatory and immunoregulatory cytokines [116], which allows viral clearance, while minimizing immune response-mediated lung damage. These observations might be particularly relevant in the context of ventilation-associated pneumonia, cytokine storm, and ARDS, all features of COVID-19. Moreover, other studies also show the probiotics’ ability to interfere with ACE2 [119,120], which might also open new avenues to a potential employment of these supplements in the management of SARS-CoV-2 infection.

Regarding prebiotics, there is much less information in the context of respiratory tract viral infections, as they are mainly used to improve gut health, according to the ISSAP. Like probiotics, synbiotics seem to be capable of reducing mechanical ventilation-associated pneumonia and repressing respiratory infections [121], but there is not much available information regarding their antiviral effects. Although there is evidence for antiviral activity of probiotic strains against common respiratory viruses, none of these effects or mechanisms have been tested on the new SARS-CoV-2 virus, despite that effects of probiotics against other coronavirus strains have been reported [122,123,124].

It is worth mentioning that not all probiotics act in the same way and have the same effects in every individual [125]. Probiotics may contain either a single strain or a mixture of strains and their effects are often strain specific [108]. In fact, the effects exerted by a strain may be different when it is administrated individually or in combination and are also variable depending on the patient group [108]. Therefore, the rationale for using probiotics in COVID-19 patients can be merely extrapolated from indirect hypothetical evidence, as there is currently no direct evidence of its effectiveness in reducing or preventing COVID-19. However, clinical trials are being performed in order to evaluate the effects of probiotics on the microbiome of household contacts exposed to COVID-19 (Clinical Trial gov Identifier NCT04399252, 2020) and their usefulness in the prevention and treatment of COVID-19 (Clinical Trial gov Identifiers NCT04366180, NCT04366089, NCT04368351). Without current science-based recommendations of prebiotics, probiotics, and synbiotics to manage COVID-19, the use of these supplements as adjunctive therapy may help to enhance host immunity and possibly better face (or prevent) the SARS-CoV-2 infection [115,116].

#### 4.2.2. Vitamins

Vitamins are micronutrients that play major functions essential to maintain body homeostasis, as they are cofactors for many enzymes, including those facilitating fat and carbohydrate metabolism. Because they cannot be synthesized in the human organism at all or in sufficient amounts (except vitamin D), they are usually obtained through the diet, namely from fruits and vegetables, but also from meat, eggs, nuts, and seeds, among other sources. However, as many vitamins are extremely sensitive to temperature and easily degraded during food processing and storage, vitamin deficiencies may occur in certain populations, namely in vegans, in elderly individuals, as well as in disease states. While low vitamin levels have been associated with increased risk of cardiovascular disease, neurodegenerative disorders, and cancer, supplementation has been reported as beneficial [126,127,128,129], which is mainly owing to its antioxidant and anti-inflammatory effects, as well as relevant metabolic and immune functions.

Vitamins play important and complementary roles in supporting the innate and adaptive immune systems [130,131]. Deficiency of certain vitamins negatively affects the immune function and may decrease resistance to infections [130,131]. Vitamins are involved in the development and maintenance of physical barriers as they influence the stratification, differentiation, and maturation of epithelial cells [130,132]. Furthermore, vitamins affect the production and activity of antimicrobial proteins, promote cytokine production, and have antioxidant properties [130,131]. Regarding the immune cells, vitamins promote their growth, differentiation, and motility, as well as death capacity and phagocytic activity in the case of neutrophils and macrophages [130,132,133]. Vitamins’ involvement in the adaptive immunity is related to their ability to promote lymphocyte differentiation, proliferation, and homing, as well as antibody production and generation of memory cells [130,132,133]. There are several lines of evidence of a reduction in the risk of respiratory infections resulting from supplementation with vitamins, including C, D, and E [130]. Therefore, vitamins might be a nutraceutical option, in combination with therapeutic strategies, to manage SARS-CoV-2 infection.

Apart the role played in the “education” and reinforcement of the immune system, GM is involved in several metabolic functions with major relevance for the host, including synthesis of certain vitamins, particularly vitamins C and K, as well as vitamins of the B group, such as folates, nicotinic acid, biotin, and cobalamin, among others [134]. Therefore, under conditions of GM dysbiosis, the synthesis of some vitamins may be impaired, which affects their metabolic and immune functions. At the same time, deficient levels of vitamins, as observed in some disease conditions, have been associated with the promotion of gut dysbiosis, as recently reviewed [135]. Mounting evidence suggests that gut microbiome dysbiosis and vitamin deficiency are deeply interconnected and that this relationship may directly impact host health in distinct conditions, including in Crohn’s disease, type 2 diabetes, and multiple sclerosis, among others. For instance, vitamin D deficiency or reduced expression of its receptor has been associated with changes in GM towards dysbiosis in some disease states, while supplementation, namely with vitamin D3, has been described as beneficial [135,136,137,138]. Curiously, intake of very high levels of vitamin D might cause harmful effects due to a reduction of bacterial richness and change of composition [139]. Other studies showed significant effects of several vitamins on GM, including vitamins A, B, C, and E [140]: vitamin A can modulate health-beneficial microbes of the genera *Bifidobacterium*, *Lactobacillus*, and *Akkermansia*; vitamin D and E modulate health-beneficial microbes of the genera *Roseburia*; vitamin D and E may reduce the F/B ratio; and supplementation of vitamins C, D, and E could modulate health-beneficial microbiota, especially favorable species from the genera *Bifidobacterium* and *Lactobacillus*. In a mice model of ileal pouchitis, vitamin E associated with selenium (Se) and retinoic acid was able to reshape the gut microflora toward an anti-inflammatory effect, thus ameliorating mucosal inflammation, which was associated with a rise in the relative percentage of *Bacteroidetes* and a decrease in *Firmicutes* [141].

Collectively, the available data point to a beneficial effect of vitamins’ supplementation on GM, which seems to depend on the concentration/dose. Finally, vitamin A has been suggested as an adjuvant therapy for infectious diseases, including viral, possibly due to GM modulation [142,143]. In fact, vitamin A supplementation (as retinoic acid) was able to inhibit norovirus replication in a murine model, which was accompanied by significantly increased abundance of *Lactobacillus*, which could be the responsible for the antiviral effect [142,143]. Further data regarding the impact of vitamin A, and others, on GM of COVID-19 patients are needed.

#### 4.2.3. Selenium and Zinc

Selenium (Se) is an essential trace mineral (non-metal) required for selenocysteine synthesis that is crucial for the production of selenoproteins, such as glutathione peroxidases (GPxs), which play a major role in redox state regulation [144]. Se strongly influences immune responses, being able to promote them and protect the organism against certain pathogens [145]. Both deficiency and excess of Se have been associated with adverse health effects often characterized by a U-shaped relationship [146]. In animal experimental studies, Se deficiency was associated with weaker immune responses against viruses, tumors, and allergens [145]. In fact, deficiency in Se affects not only immune function, but also the viruses themselves. Oxidative stress resulting from Se deficiency might cause alterations in the viral genome that could increase its virulence [131,145], as showed for influenza A virus, which suffers RNA mutations because of Se deficiency [147]. When compared with mice with normal levels, Se-deficient mice infected with the influenza virus showed a decrease in macrophages and CD4+ and CD8+ T cells numbers [147]. This suggests that Se levels influence cell-mediated-immunity. Additionally, individuals infected with HIV-1 with reduced Se concentrations present a lower number of CD4+ T cells, higher disease progression, as well as higher disease-related mortality [145]. Evidence showed that the synergistic effect of Se with ginseng stem-leaf saponins might induce a strong immune response in a vaccine against bivalent infectious bronchitis coronavirus in chickens [131]. In addition, mounting evidence shows that Se supplementation (in moderate doses) can affect GM composition, increasing the diversity and modulating a broad range of microbes. In particular, Se has been associated with reduced levels of deleterious microbes, such as *Dorea* and *Mucispirillum*; augmented levels of beneficial microbes, such as *Akkermansia, Lactobacillus*, and *Faecalibacterium*; as well as enriched concentration of short chain fatty acids (SCFAs), namely butyric acid [140,148]. The beneficial effects of Se in models of inflammatory bowel diseases and experimental colitis have been attributed to the symbiotic properties towards a healthier intestinal microbiota [149,150].

Zinc (Zn) is the second most abundant dietary essential trace mineral (metal) in the human body after iron, which plays a major role in the immune system cells’ maintenance and development [151]. The presence of Zn is essential to the normal development and function of cells belonging to the innate and adaptive immune system, NK cells, neutrophils, macrophages, and T and B cells [152]. Zn deficiency affects many organs, having a particular impact on the immune system, which is prominently susceptible to changes of Zn levels [153]. Adequate levels of Zn ensure phagocytosis events and cytokine production, which could be crucial for the immune response against infections (bacterial and viral) and inflammatory diseases [152,153]. In fact, increasing the intracellular Zn concentration using ionophores can inhibit the replication of several RNA viruses [154]. Interestingly, it was recognized that a combination of Zn and the ionophore pyrithione at low concentrations prevents SARS-CoV replication [154]. In preclinical studies using distinct models, Zn deficiency has been associated with significant taxonomic alterations and decreased overall species richness and diversity, thus causing a microbial profile similar to what is found in several pathological conditions [155]. In addition, Zn supplementation has been associated with a reduction of deleterious microbes and a rise of beneficial ones [140]. However, the beneficial effects seem to be dose-dependent. In fact, evidence showed that excess dietary Zn is able to impair GM, decreasing microbial diversity and promoting a significant shift in community structure; in addition, exacerbated C. difficile infection caused a reduced threshold of antibiotics needed to confer susceptibility to the infection [156].

Taken together, the available data suggests that Se and Zn supplementation (at non-toxic levels), through their ability to modulate GM towards symbiosis and to strengthen the immune system, might have beneficial effects against infections and could be further tested as adjunctive measures against SARS-CoV-2 infection. Thus, further research in COVID-19 patients is recommended.

#### 4.2.4. Flavonoids

Flavonoids are products of plants’ secondary metabolism and include about 6000 phenolic compounds [157] that display polyphenolic structure responsible for the antioxidant properties [157]. However, their biologic functions go beyond the well-recognized antioxidant effects, extending to anti-inflammatory and immune-modulation properties, which might contribute to explain the protective effects described in a broad range of cardiovascular, metabolic, and neurodegenerative disorders [158,159,160]. Different flavonoids have been also investigated in vitro and in vivo regarding their antiviral properties [161]. For example, luteolin and luteolin-rich fractions have shown antiviral properties against SARS-CoV, rhesus rotavirus, chikungunya virus (CHIKV), and Japanese encephalitis virus [161]. On the other hand, flavonol Kaempferol derivates have demonstrated a strong inhibitor effect in the 3a channel of coronavirus, which is involved in the virus releasing mechanism [161]. One of these derivates, kaempferol-3-O-α-L-rhamnopyranoside, obtained from Zanthoxylum piperitum, was able to inhibit the replication of influenza A virus in vitro [161]. Additionally, Yu et al. discovered that myricetin might inhibit SARS-CoV infection by viral helicase’s ATPase activity interference [162]. Jo et al. also suggested that the antiviral activity against coronavirus of some flavonoids may rely on the 3C-like protease (3CL pro) inhibition [163].

Apart from a potential interference with virus replication (including SARS-CoV), flavonoids as well as other polyphenols have the ability to modulate GM [20]. The effects have been mainly described as promoters of growth of some symbiotic microbes, such as *Bifidobacterium* spp. and *Lactobacillus* spp, while reducing pathogenic microorganisms, such *Clostridium* spp., *Helicobacter pylori, Escherichia coli,* and *Salmonella typhimurium* [20,164]. Furthermore, flavonoids can also improve gut health by reducing the production of endotoxins, by regulating intestinal immune homeostasis, and by promoting adequate absorption of nutrients [165]. Those effects encouraged the use of flavonoids towards the prevention and/or treatment of intestinal and extra-intestinal diseases, such as inflammatory bowel disease and obesity, among others [164,166]. In addition, flavonoid–microbiome interactions were found to be helpful in the treatment of viral infections in two distinct studies with virus influenza involving bacterial processing of flavonoids [167,168]. Additional studies, namely in COVID-19 patients, are required.

#### 4.2.5. Omega-3 Polyunsaturated Fatty Acids

Omega-3 polyunsaturated fatty acids (ω-3 PUFAs) are the main components of fish and flaxseed oils [169]. Several in vitro and in vivo studies have described ω-3 PUFAs’ effects on the immune system [169,170,171,172]. They are able to influence various macrophages’ activities, such as cytokine and chemokine production and secretion, phagocytosis, and polarization, to classically-activated or alternatively-activated macrophages [169]. Additionally, these fatty acids are incorporated in phospholipids of neutrophils’ cell membrane and can be metabolized by these cells into prostaglandins, leukotrienes, thromboxanes, maresins, protectins, and resolvins, which are immune-regulatory metabolites [169]. ω-3 PUFAs are also associated with enhanced activation of T and B cells [169]. Morita et al. demonstrated that the ω -3 polyunsaturated fatty acid lipid mediator D1 (PD1) inhibits the infection by influenza A virus in cell culture [173]. PD1 acts at the genetic expression level, preventing the exportation of viral RNA by inhibiting its interaction with the nuclear export factor NXF1. PD1 administration in mice infected with influenza A virus showed a decrease in the viral load and improved survival [173], thus suggesting that ω-3 PUFAs might have therapeutic potential. However, there are also studies that suggest the opposite. For example, it was reported that, in murine influenza, ω-3 PUFAs’ supplementation results in a delayed clearance of the virus, decrease in interferon γ (IFN-γ) and immunoglobulin A production, and cytotoxicity suppression of lung T cells [174].

Mounting evidence coming from animal models strongly suggest that the crosstalk between ω-3 PUFAs, GM, and the immune system could be crucial to maintain intestinal wall integrity and reinforce host immune cells towards a better response against infections [175]. Preclinical research, and one clinical study, have shown a beneficial effect of dietary ω-3 PUFA intake (or high tissue levels) in intestinal microbiota, viewed by an increased amount of certain SCFA (butyrate)-producing beneficial genera, namely *Bifidobacterium, Lachnospira, Roseburia*, and *Lactobacillus* [176,177]. However, further investigation is required in order to determine if ω-3 PUFA could be a relevant adjunctive strategy against SARS-CoV-2 infection.

#### 4.2.6. Traditional Chinese Medicine

More than 85% of SARS-CoV-2 infected patients in China received treatment with Traditional Chinese Medicine (TCM), which was supported by the promising beneficial effects found against SARS-CoV [178]. Lau et al. performed a study during the SARS-CoV outbreak where 1063 volunteers, which included 962 hospital workers and 37 laboratory technicians that worked in high risk laboratories, were treated with a TCM herbal extract composed of Sang Ju Yin and Ping Feng San [179]. When compared with the control group, (with an infection percentage of 0.4%), none of the TCM users were infected. Cinatl et al. demonstrated that glycyrrhizin, a component of liquorice roots, is able to inhibit the replication of SARS-CoV-associated coronavirus in vitro [180]. Recently, it was predicted that this compound might be able to bind to ACE2 receptors, thus having potential effects against SARS-CoV-2 infection [98]. It was also reported that hesperin, a natural flavonoid found in citrus fruits, has potential to block SARS-CoV-2 link to ACE2 [98]. Baicalin, a flavone isolated from Scutellia baicalensis, showed antiviral properties against SARS-CoV [81]. Another flavone, quercetin, has shown antiviral effects by inhibiting SARS-CoV 3CL protease and blocking its entrance into the host cells [81,181,182]. More recently, the recovery of 50 out of 51 COVID-19 patients treated with TCM in combination with classical antiviral and anti-inflammatory drugs was described, including interferon, lopinavir, ritonavir, and short-term corticosteroids therapy [183].

Accumulating evidence shows that TCM could interfere with GM [184]. Changes in composition and diversity using TCM could be achieved by interference with gastrointestinal pH and transit time, as well as by affecting GM enzymes and metabolites [185]. In addition, some TCM compounds (such as Xiexin Tang and other herbs) can reshape the GM in T2DM rats, viewed by increased abundance of bacteria with the ability to synthesize SCFAs and with anti-inflammatory properties [186]. Similar effects were observed in diabetic patients taking Gegen Qinlian decoction (GQD, traditionally prescribed for hyperglycemia), which was associated with reduced abundance of *Faecalibacterium prausnitzii* [187]. Gut dysbiosis in obese mice induced by high-fat diet was reversed by the water extract of ganoderma lucidum mycelium, with a decreased *Firmicutes–Bacteroidetes* ratio and endotoxin-bearing *Proteobacteria* levels [188]. Similar effects have been described for a broad range of TCM compounds [184,189]. Therefore, TCM might be considered an option for management of COVID-19, most probably in combination with therapeutic options. However, the mechanisms underlying its antiviral action need to be further dissected and consistent information from SARS-CoV-infected patients is required.

### 4.3. Prophylactic Approaches

#### 4.3.1. BCG Vaccination

Bacillus Calmette-Guérin (BCG) is a live attenuated vaccine against tuberculosis that was developed in the Pasteur Institute in 1921 [190,191]. The original BCG vaccine was distributed to laboratories around the world and maintained by serial passages. According to the distribution period, these strains can be classified as “early strains” and “late strains” [190]. Since then, it has been the most administrated vaccine worldwide, with near 130 million children vaccinated every year [191].

Epidemiological studies report that BCG vaccination significantly reduced infant mortality, which cannot be explained by a decrease in tuberculosis alone [191]. In fact, besides the protective effect against tuberculosis, the BCG vaccine exerts heterologous immunologic effects, offering protection against other infectious agents and, specially, respiratory tract infections [191,192,193,194,195,196,197]. Cellular and molecular mechanisms responsible for the beneficial effects of BCG vaccination against viral infections are associated with metabolic and epigenetic modifications that prime the innate immune response to subsequent infections, a process referred to as “trained immunity” [190,191,193]. Vaccination of human healthy volunteers resulted in an increased production of pro-inflammatory cytokines when monocytes were stimulated with distinct pathogens ex vivo [190,191,193]. These effects were accompanied by histone modifications and reprograming of promoter regions belonging to inflammatory cytokines-codifying genes [190,191].

Ecological epidemiological studies suggest that regions that have or had a mandatory BCG vaccination program present a lower number of SARS-CoV-2 infected people, as well as an inferior mortality from COVID-19 [191]. Seven of eight countries with a very low number of total deaths have adopted an obligatory BCG vaccination program [190]. It was observed that this putative protective effect was still present in 14-year-old children, suggesting that BCG vaccination effects might be long-lasting [198]. The apparent lack of protection from BCG vaccine against COVID-19 in France and in the United Kingdom, where the vaccine was administrated to older children, suggests that trained immunity might not occur in older children or might have a shorter extent [198]. It is also possible that the strains used and the administration mode affect the response to vaccination [198]. On the other hand, in many countries where BCG vaccination was interrupted or discontinued, mortality from COVID-19 is much higher [190]. The same also holds true for some countries that had used BCG Denmark strain [190].

BCG strains that seem to be associated with lower mortality from COVID-19, such as BCG Japan and BCG Russia, are early strains, while BCG Denmark, which seems less protective against the disease, is a late strain [190]. Several studies demonstrated that these strains differ in phenotypic and genetic aspects. It was demonstrated that late strains evolved from the early ones through genetic mutations that might have led to the loss of some proteins expression, including MPB64, MPB70, and MPB83 [190]. Another study demonstrated that early strains have much higher bacterial counts, suggesting that these might be richer in immune responses-stimulating substances when compared to late strains [190]. However, evidence from Finland and Australia appears to contradict the hypothesis that early strains give more protection against COVID-19, because, even though these countries have ceased BCG vaccination years ago, they still present low mortality from the disease when compared with countries with mandatory BCG vaccination [190]. These results suggest that BCG vaccination is not the only factor contributing to the observed lower mortality. In fact, both countries have excellent healthcare systems and low population density, which might reduce disease dissemination [190].

Even though there are several studies suggesting a protective role of BCG vaccination against SARS-CoV-2 infection, these cannot provide definitive proofs of causality. Accordingly, a huge number of variables deserve particular attention, namely, the differences in population demography and genetics, in non-pharmaceutical interventions, in diagnosis and accurate reporting of COVID-19 cases, and in pandemic severity across distinct countries and regions [191,198,199]. A recent study presented a refined epidemiological analysis to mitigate the effects of important confounding factors (such as stage of the COVID-19 epidemic, development, rurality, population density, and age structure) [200]. The authors obtained a correlation between BCG vaccination index and COVID-19 mortality in some European countries with identical social profile; for every 10% increase in the BCG index, there was a 10.4% reduction in mortality. Despite these data, an association between BCG vaccination and mortality from COVID-19 remains a hypothesis that deserves to be better tested in the future.

The immune response to vaccination is strongly influenced by the host’s immune system [201,202]. As the GM is important in the regulation of immune responses, it might influence responses to vaccines as well. Therefore, changes in microbiota’s composition may have an impact on the immune response resulting from vaccine administration [201,202,203]. Observed heterogeneity in vaccine response across the population might in fact be partially derived from variations in microbial composition resulting from different conditions regarding nutrition, hygiene, environment, and socioeconomic status [201]. Both the microbial community and the immune system develop during the first months of life, which is coincidental with the period in which most vaccines are administrated [201]. Consequently, early microbiota composition might have an influence in vaccine responses.

Evidence demonstrates that GM plays an important role in the response to vaccination, including pre-clinical and clinical studies. These studies show that GM constitutes a source of natural adjuvants that are capable of activating different mechanisms that control innate and adaptive immunity [201]. This effect was observed with the influenza vaccine, where germ free mice and mice treated with antibiotics (Abx) showed diminished antibody responses to vaccination. An oral reconstitution with Escherichia coli was enough to restore the normal antibody response. Hence, antibiotic-driven dysbiosis may lead to impaired immune response to vaccination. In fact, it was recently demonstrated that antibiotic-driven dysbiosis in the early life of mice leads to weakened antibody responses to five vaccines frequently administrated to infants, including tuberculosis vaccine BCG [201]. In all cases, restoration of commensal microbiota reverted antibody responses’ impairment.

It is clearly known that intestinal microbiota composition imbalance increases the host’s susceptibility to tuberculosis [202]. A study where Abx was administrated to mice to elicit microbial dysbiosis revealed that Abx-treated animals displayed a decrease in microbial number and diversity when compared with untreated BCG vaccinated animals [202]. In addition, Abx mediated gut dysbiosis in BCG vaccinated animals was associated with an impaired production of effector and memory T lymphocytes, as well as with a decrease in IFN-γ and TNF-α expression [204]. These observations indicate that dysbiosis might be one of the factors responsible for the impaired immune response to BCG in a vaccinated population. The above observations might explain the variable efficacy of BCG vaccination across different countries, as GM is variable among populations. In another study, beyond T cells-mediated immune response impairment, there was also significant weakening of antigen-specific IgG responses in mice vaccinated with BCG and treated with Abx [205]. In addition, Huda et al. (2014) analyzed stool microbiota of 48 Bangladeshi infants and found that immune response to BCG vaccine was positively correlated with the abundance of *Bifidobacterium*. Individuals with high levels of *Bifidobacterium* demonstrated a higher level of adaptive immune response to vaccination. On the other hand, the opposite effect is observed in individuals with high levels of *Enterobacteriales, Pseudomonadales*, and *Clostridiales* [206]. These results suggest that microbiota composition and diversity might be crucial in promoting immune responses after vaccination, and these might be enhanced by intestinal *Bifidobacteria* promotion and by minimizing dysbiosis in the infancy.

Therefore, it is important to consider the role of the intestinal microbiota in immune responses and, consequently, in the efficacy of different vaccines, including BCG. Further knowledge is needed regarding this association in order to understand and explain the observed heterogeneity of responses to the same vaccine and to potentially maximize vaccination efficacy through microbiota modulation.

#### 4.3.2. New Vaccines for SARS-CoV-2

In addition to the variety of therapeutic and nutraceutical approaches that have been tested to treat people with COVID-19 and/or minimize its consequences, researchers’ teams from Universities and R&D Institutes from different parts of the world, supported by pharmaceutical and biotechnological companies, have been making a huge effort to develop an effective (and safe) vaccine against the disease [207,208,209]. This is a true run against time that promises to surpass all the deadlines that we acknowledge as minimums to guarantee the necessary validation in terms of efficiency and safety over the course of various phases, from pre-clinical tests to clinical trials. The biotechnological advance, together with some existing knowledge regarding coronaviruses that previously caused human diseases (SARS-CoV and MERS), as well as the urgency of more efficient bureaucratic procedures, may allow to remarkably shorten the normal deadlines for developing a vaccine (over 10 years) to 12–18 months, if the current expectations will be further confirmed [207].

There are seven main platforms (categories) used for vaccine development [207,208,209]. These include two nucleic acid platforms (DNA and RNA) that belong to the new generation of vaccines, which could be further sub-divided according to delivery and carriers. A “protein-based” or “subunit” category, which includes various technologies to prepare immune-stimulating viral protein antigens; this category comprises the majority of COVID-19 vaccine candidates. Two categories that make use of viral-based vectors, namely non-replicating and replicating vectors, and two others that are the SARS-CoV-2 viruses themselves, either inactivated or in a live-attenuated version. The “other” category, includes other strategies, such as virus-like particles and the use of non–SARS-CoV-2 virus carriers, among others. Finally, there are approaches based on repurposing of existing vaccines (for polio or tuberculosis, as examples) to evoke general immunity.

Of the more than 150 projects of development of COVID-19 vaccine candidates, about a dozen are already in more advanced stages of clinical trials, thus presenting more possibilities to achieve results eventually compatible with approval for mass production and use in humans [207,208,209]. These include the following, which are already in phase 3 trials: ChAdOx1/AZD1222, from University of Oxford/AstraZeneca (NCT04400838 and NCT04516746); mRNA-1273, from Moderna TX Inc./National Institute of Allergy and Infectious Diseases (NCT04470427); Ad5-nCoV, from CanSino Biologicals Inc./Beijing Institute of Biotechnology (NCT04526990); PiCoVacc, from Butantan Institute/Sinovac Life Sciences Co. Ltd. (NCT04456595); BNT162, from BioNTech/Pfizer (NCT04368728); Vero Cell, from China National Biotec Group (NCT04510207); and Ad26.COV2.S, from Janssen Vaccines & Prevention B.V. (NCT04505722).

The results of these clinical trials are awaited with high expectations and with the hope that at least one of these vaccines in development (but preferably more) will show the necessary efficacy (and safety) in populations with different ages and genetic/ethnic backgrounds. It is known that vaccine-induced immune responses are extremely variable between individuals/populations of different world regions. Several factors are able to determine intra- and inter-population variations in vaccine responses, including host genetics, nutritional status, and immunological imprinting, among others. Mounting evidence points to a chief role of the GM composition and function in the immune responses to vaccination [210]. In this context, it will be interesting to understand how the response to the vaccine(s) for COVID-19 may or may not be influenced by GM.

## 5. Concluding Remarks and Perspectives

In the last century, infection due to SARS-CoV-2 is already the third caused by coronavirus in humans, having reached pandemic proportions, putting significant pressure on the health systems of different countries, including the most developed ones, and causing a huge number of deaths, which continue to rise. Although COVID-19 is asymptomatic in most infected people, it progresses from moderate symptoms (such as fever, cough, fatigue, shortness of breath, and headache) to a more complex respiratory condition that, in more severe cases, causes complications in several organs and systems, including cardiovascular, renal, and even central nervous system, which can culminate in death. Although some factors and populations with a higher risk of poor prognosis are already known, such as the elderly, especially with pre-existing comorbidities, particularly at the cardiovascular, metabolic, and respiratory levels, the difficulty in predicting who and how will evolve to a more severe clinical picture and who and why will recover from a situation of greater severity continues to challenge the medical and scientific community. Among other possible factors, the great pathophysiological complexity of the disease can play a decisive role. Indeed, it is known that the infection can easily extend from the respiratory tract to other systems, including the gastrointestinal, triggering an exacerbated immune response that leads to severe and eventually persistent inflammation, which hardly could be resolved, especially in situations accompanied by a weak immune system, such as those associated with some elderly populations and pre-existent diseases.

**Figure 1:**
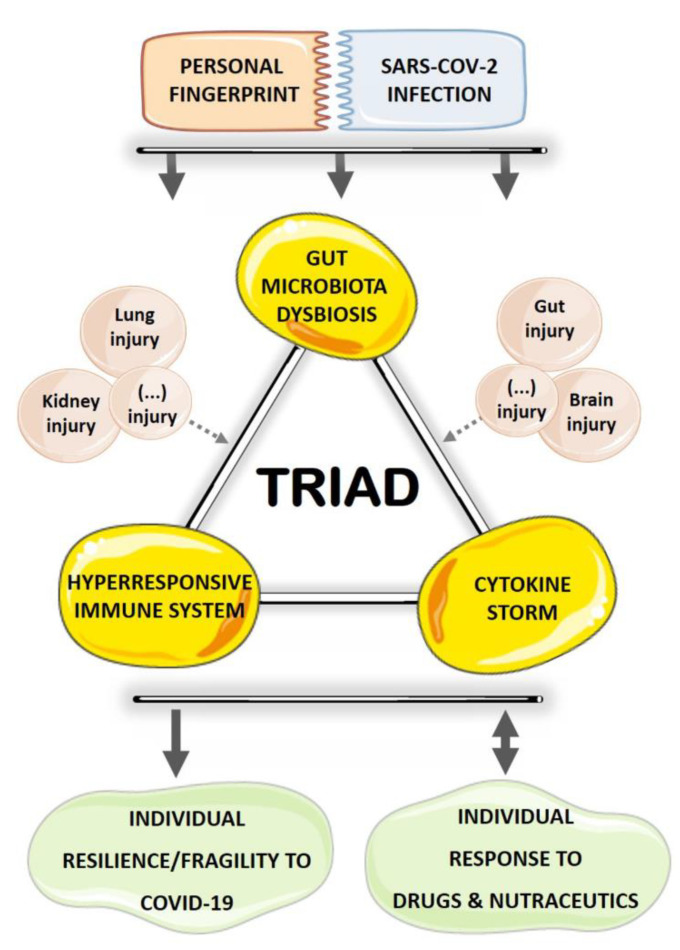
The interplay between the gut microbiota dysbiosis–immune hyperresponse–inflammation triad in Coronavirus Disease 2019 (COVID-19) and the putative influence on disease progression and response to therapies. Rather than being merely restricted to the lower respiratory tract, severe acute respiratory syndrome coronavirus 2 (SARS-COV-2) infection extends to other organs, namely the gastrointestinal tract (multi-tissue infection). Influenced by the lifetime cross-modulations between the immune system and the microbiota (like a personal fingerprint), gut microbiota dysbiosis, immune hyperresponse, and an inflammatory setting are elicited under these circumstances (triad). On behalf of the intricate influence of gut microbiota (GM) on host immune effectors and subsequent inflammatory profile, GM composition and function might contribute to explain the individual resilience/fragility to COVID-19 and/or the response to therapeutics, which deserves further research.

GM is among the most important factors for the education and strengthening of immune response to fight against infections, including viral infections. In fact, intestinal microflora and the immune system are in constant association, modulating each other throughout the entire life in order to maintain the host’s homeostasis, thus creating a kind of a personal fingerprint. However, GM has been also described as being impaired (dysbiosis) in ageing people and in individuals with cardiovascular, metabolic, and kidney diseases. In addition, recent evidence also points to an imbalance of GM in patients with COVID-19. Thus, the GM dysbiosis–immune hyperresponse–inflammation triad, influenced by the personal fingerprint, could eventually explain why some COVID-19 patients are more resilient while others are more fragile when infected with SARS-CoV-2, recovering faster or progressing to more a severe clinical condition (Figure 1, left). In addition, it is known that many therapeutic interventions that have been tested can influence the three components of the triad in general, eventually in a synergistic fashion, and GM in particular. In fact, several pharmacological and nutraceutical approaches, and even BCG vaccination, present clear immunomodulatory and anti-inflammatory properties, as well as the ability to interfere with GM; in addition, the triad (but in particular GM) could also affect therapeutics’ efficacy, namely by interfering with the bioavailability and other pharmacokinetic parameters of drugs and compounds. So, the personal fingerprint could also eventually contribute to explaining why some COVID-19 patients respond better to some of the therapeutic and nutraceutical interventions that have been tested while other are poorly responsive, thus suffering disease aggravation (Figure 1, right). The crosstalk between drugs and food or natural components and the components of the triad, particularly with GM, has been mainly described in other pathological conditions, but merits further investigation in COVID-19 patients. This knowledge can be pivotal to unveil the approaches with greater possibility of success for specific populations regarding the treatment of COVID-19, in a logic of personalized/individualized medicine.

## Figures and Tables

**Table 1:** Clinical trials with COVID-19 patients for the study of gut microbiota (Clinicaltrial.gov).

ID, Country, and Status ^†^	Study Type and Participants	Trial Title (Main Hypothesis/Aims)
NCT04325919 Hong Kong Recruiting	Obs./Prosp. Hospitalized COVID-19 patients (170 *)	Comprehensive Clinical, Virological, Microbiological, Immunological and Laboratory Monitoring of Patients Hospitalized With COVID-19
NCT04359706 France Recruiting	Obs./Prosp. COVID-19 patients at ICU (30 *)	Bacterial and Fungal Microbiota of Patients with Severe Viral Pneumonia With SARS-CoV2 (determine the respiratory and fecal microbiota—microbial and fungal—of critically ill patients)
NCT04410263 Switzerland Recruiting	Obs./Prosp. COVID-positive patients at ICU (300 *)	Microbiota in COVID-19 Patients for Future Therapeutic and Preventive Approaches
NCT04486482 USA Recruiting	Interv./Par. Ass. Mild-to-moderate COVID-19 patients (50 *)	An Exploratory, Open Label, Clinical Study to Evaluate the Physiologic Effects of KB109 in Adult Patients with Mild-to-Moderate COVID-19 on Gut Microbiota Structure and Function in the Outpatient Setting
NCT04399252 USA Recruiting	Interv./Par. Ass. Exposed household contacts of COVID-19 (1000 *)	A Randomized Trial of the Effect of Lactobacillus on the Microbiome of Household Contacts Exposed to COVID-19
NCT04355741 Portugal Recruiting	Obs./Prosp. COVID-19 patients (60 *)	Gut Microbiota, “Spark and Flame” of COVID-19 Disease (fecal gut microbiota composition could affect vulnerability and disease outcomes of COVID-19)
NCT04366089 Italy Recruiting	Interv./Par. Ass. Hospitalized COVID-19 patients (152 *)	Oxygen-Ozone as Adjuvant Treatment in Early Control of Disease Progression in Patients With COVID-19 Associated with Modulation of the Gut Microbial Flora (Phase 2)
NCT04332016 France Recruiting	Obs./Prosp. Hospitalized COVID-19 patients (2000 *)	COVID-19 Biological Samples Collection (COLCOV19-BX)
NCT04458519 Canada Not yet Recruiting	Interv./Par. Ass. COVID-19 patients not requiring hospitaliz. (40 *)	Randomised Single Blinded Clinical Study of Efficacy of Intranasal Probiotic Treatment to Reduce Severity of Symptoms in COVID19 Infection
NCT04327570 Belgium Recruiting	Obs./Prosp. Hospitalized COVID-19 patients (100 *)	In-depth Characterisation of the Dynamic Host Immune Response to Coronavirus SARS-CoV-2 (Correlation of immune profiling with microbiome analysis)
NCT04390477 Spain Recruiting	Interv./Par. Ass. Hospitalized COVID-19 patients (40 *)	The Intestinal Microbiota as a Therapeutic Target in Hospitalized Patients With COVID-19 Infection (a positive effect of probiotic on the GM that could produce a less severe clinical evolution of the disease)
NCT04368351 Italy Active, not recruiting	Obs./Retros. Hospitalized COVID-19 patients (70 *)	Evaluation of the Impact of Bacteriotherapy in the Treatment of COVID-19 (probiotic supplementation (SivoMixx) + Azithromycin)
NCT04373148 USA Recruiting	Obs./Prosp. Adults and children with COVID-19 (1000 *)	Understanding Immunity to SARS-CoV-2, the Coronavirus Causing COVID-19
NCT04451577 Italy Recruiting	Obs./Case-Control Hospital employees with or without COVID-19 (5000 *)	Epidemiologic, Clinical, Molecular Characteristics of Hospital Employees With or Without Covid-19 Infection: a Retrospective-prospective Cohort Study
NCT04497402 Italy Not yet Recruiting	Obs./Prosp. Covid-19 patients (88 *)	Sex-Informed Data in the COVID-19 Pandemic (determine whether there are sex differences in biomarkers, including in gut microbiome)
NCT04359459 The Netherlands Not yet recruiting	Obs./Prosp. COVID-19 patients (150 *)	Nasal CIliated EPithelial Genetic and Single Cell RNA prOfiLes of miLd, Severe and Very Severe COVID-Nineteen patIents (CIPOLLINI) Study (correlation of feces microbiome and clinical outcome for COVID-19)
NCT04403646 Argentina Not Yet Recruiting	Interv./Par. Ass. Hospitalized COVID-19 patients (140 *)	Efficacy of Tannin Specific Natural Extract for Coronavirus Disease (COVID-19): Randomized Controlled Trial
NCT04517422 Mexico Not Yet Recruiting	Interv./Par. Ass COVID-19 patients with mild symptoms (300 *)	Efficacy and Safety of Lactobacillus Plantarum and P. Acidilactici as Co-adjuvant Therapy for Reducing the Risk of Severe Disease in Adults With SARS-CoV-2 and Its Modulation of the Fecal Microbiota: A RCT
NCT04447144 Egypt Recruiting	Obs./Prosp.; COVID-19 patients with mild and moderate severity (200 *)	Nutritional Habits, Does it Affect Coronavirus Disease 2019 (COVID-19) Infection Outcome? An Egyptian Experience
NCT04420676 Austria Not yet recruiting	Interv./Par. Ass. COVID-19 patients (108 *)	Synbiotic Therapy of Gastrointestinal Symptoms During Covid-19 Infection: A Randomized, Double-blind, Placebo Controlled, Telemedicine Study (SynCov Study) (probiotic supplementation: Omnibiotic^®^ AAD)
NCT04345510 Germany Not yet recruiting	Obs./Prosp. Asymptomatic Carriers (500 *)	Testing for COVID-19 Infection in Asymptomatic Persons (analyze for a possible correlation between oral microbiome and COVID-19 infection status)
NCT04444609 United Kingdom Recruiting	Obs./Prosp. COVID-19 patients with lung disease (230 *)	PROSAIC-19 - Prospective Longitudinal Assessment in a COVID-19 Infected Cohort
NCT04359836 USA Recruiting	Obs. COVID-19 patients (250 *)	A Non-Interventional Pilot Study to Explore the Role of Gut Flora in COVID-19 Infection

COVID-19, Coronavirus disease; ICU, intensive care unit; Interv., interventional; Obs., observational; Par. Ass., parallel assignment; Prosp., prospective; GM, gut microbiota. ^†^ Except those indicated, all the trials are Phase “Not Applicable”, which is a definition used to describe trials without Food and Drug Administration (FDA)-defined phases. * Estimated total number of participants.
